# Development of a Prognostic Model That Predicts Survival After Pancreaticoduodenectomy for Ampullary Cancer

**DOI:** 10.1097/MPA.0000000000000929

**Published:** 2017-09-12

**Authors:** Michael Feretis, Tengyao Wang, Satheesh Iype, Adam Duckworth, Rebecca Brais, Bristi Basu, Neville V. Jamieson, Emmanuel Huguet, Anita Balakrishnan, Asif Jah, Raaj K. Praseedom, Simon J. Harper, Siong-Seng Liau

**Affiliations:** From the *HPB Unit and University Department of Surgery, Addenbrooke's Hospital; †Statistical Laboratory, Department of Statistics, University of Cambridge; and Departments of ‡Histopathology, §Oncology, Addenbrooke's Hospital, Cambridge, UK.

**Keywords:** ampullary adenocarcinoma, periampullary tumors, pancreaticoduodenectomy, survival

## Abstract

**Objectives:**

The aims of this study were to (i) identify independent predictors of survival after pancreaticoduodenectomy for ampullary cancer and (ii) develop a prognostic model of survival.

**Methods:**

Data were analyzed retrospectively on 110 consecutive patients who underwent pancreaticoduodenectomy between 2002 and 2013. Subjects were categorized into 3 nodal subgroups as per the recently proposed nodal subclassification: N0 (node negative), N1 (1–2 metastatic nodes), or N2 (≥3 metastatic nodes). Clinicopathological features and overall survival were compared by Kaplan-Meier and Cox regression analyses.

**Results:**

The overall 1-, 3-, and 5-year survival rates were 79.8%, 42.2%, and 34.9%, respectively. The overall 1-, 3-, and 5-year survival rates for the N0 group were 85.2%, 71.9%, and 67.4%, respectively. The 1-, 3-, 5-year survival rates for the N1 and N2 subgroups were 81.5%, 49.4%, and 49.4% and 75%, 19.2%, and 6.4%, respectively (log rank, *P <* 0.0001). After performing a multivariate Cox regression analysis, vascular invasion and lymph node ratio were the only independent predictors of survival. Hence, a prediction model of survival was constructed based on those 2 variables.

**Conclusions:**

Using data from a carefully selected cohort of patients, we created a pilot prognostic model of postresectional survival. The proposed model may help clinicians to guide treatments in the adjuvant setting.

Adenocarcinomas of the ampulla of Vater are relatively rare gastrointestinal malignancies accounting for approximately 0.2% of all neoplasms arising from the gastrointestinal tract.^1^ They are, however, the second most frequently encountered tumors among all periampullary neoplasms accounting for 7% to 12.7% of all periampullary neoplasms.^2,3^ Ampullary cancer, unlike pancreatic neoplasms that are most frequently diagnosed in advanced stages, usually presents with symptoms of biliary obstruction early in its development, and therefore higher resectability rates (up to 82.1%) have been reported.^2,4,5^

The definition of what constitutes an “ampullary neoplasm” has not been consistent in the literature, and questions have been previously raised with regards to considering ampullary carcinomas as a distinct tumor entity.^6^ The term “periampullary tumors” has been previously used to refer to any cancerous lesion arising from the ampulla or its immediate vicinity, and it is amenable to curative resection by means of pancreaticoduodenectomy.^7,8^ Previous reports on “periampullary tumors” have identified several histopathological or preoperative factors (tumor histologic grade, histological subtype, surgical resection margin involvement, preoperative serum bilirubin levels, and lymph node [LN] involvement) as predictors of survival after pancreaticoduodenectomy.^2,9–21^ Furthermore, recent evidence suggests that the currently used nodal staging system for ampullary tumors (N0/N1) overestimates survival in subjects with more advanced nodal disease.^9,11^

Previous reports, with the aforementioned limitations in definitions, have estimated survival after pancreaticoduodenectomy for ampullary cancer to range from 33% to 61%,^9,22^ making adjuvant therapy relevant in the context of this malignancy. However, despite identifying predictors of survival, the selection criteria for adjuvant therapies along with the survival benefit of such treatments for ampullary cancer are not clear unlike the case of pancreatic cancer where such therapies have an established role in the management algorithm.^23^ Identifying patients with high-risk features would be desirable because it would guide clinicians on offering adjuvant therapies.

The aims of this study were (i) to assess the overall survival and identify predictors of survival after pancreaticoduodenectomy for ampullary cancer in a high-volume hepatopancreatobiliary unit and (ii) to develop a pilot prognostic model that can assist clinicians in selecting patients for adjuvant therapies in the future.

## MATERIALS AND METHODS

### Patient Population

The records of 384 patients who underwent pancreaticoduodenectomy for periampullary lesions in our institution from January 2002 to December 2013 were reviewed retrospectively, with the aim of identifying those subjects who had surgery for neoplasms arising directly from the ampulla of Vater. The study was conducted after obtaining approval from our institution's patient safety and clinical governance review board.

### Selection Criteria

Patient exclusion criteria were the following: metastatic or unresectable disease at the time of surgery, indolent tumor types (islet cell, mucinous cystadenomas), ampullary neuroendocrine tumors, and tumors arising from anatomical locations adjacent to the ampulla (head of pancreas, duodenum, distal common bile duct). After applying the exclusion criteria previously mentioned, 111 patients met the study's inclusion criterion of histologically confirmed ampullary adenocarcinoma.

### Pathologic Examination of the Primary Tumor

To define the origin of the resected neoplasm, we adhered to the definition of ampullary carcinoma, as described by the Royal College of Pathologists in the UK.^24^ This is defined as a tumor grossly appearing to arise either in the ampulla of Vater (the confluence of the pancreatic and common bile ducts) or the papilla of Vater (the duodenal papilla). In cases of large tumors where the exact origin of the tumor was difficult to extract, the tumor was regarded as ampullary if it was centered in the ampulla or the duodenal papilla. The tumor subtype was determined by means of immunocytochemistry. Cytoplasmic immunoreactivity for cytokeratin 7 and cytokeratin 20 and nuclear staining for caudal-type homeodomain transcription factor 2 were assessed as per our standard institutional protocol for neoplasms of hepatobiliary origin. Data extracted from patient histology records included the tumor size, histological subtype, degree of tumor differentiation, number of LNs retrieved, number of metastatic LN, vascular invasion, perineural invasion, and surgical resection margin involvement. The histopathology findings were described according to the TNM staging system. The nodal stage was recalculated by 2 authors (M.F., S.I.) based on the revised nodal substaging protocol, proposed by Kang et al^11^ (LN negative [N0], 1–2 LN positive [N1], ≥3 LN positive [N2]). Specimens with evidence of 1 mm or smaller tumor, vascular, perineural, or nodal involvement at the resection margin, were considered to be R1 resections, as per the Royal College of Pathologists' guidelines on reporting histological outcomes after major pancreatic resections.^24^ Perineural invasion was defined as the presence of tumor cells in the space immediately surrounding a nerve. Lymphovascular (or vascular) invasion was defined as the presence of tumor within an endothelial lined or lymphatic space. Data were analyzed for all resections irrespective of LN harvest. Additional analysis was performed in those resections with 12 or more nodes retrieved.

### Statistical Analysis

Details on patient follow-up were retrieved from an institutional electronic database. The follow-up period was defined as the time interval between the date of resection and that of the last clinical interaction. Patient survival status was obtained from the UK Cancer Registry database. Because of the difficulty in differentiating cancer-related deaths from deaths from other causes, only overall survival (ie, death from all causes) was calculated. The date used for censoring during survival analysis was February 1, 2015.

Continuous variables are reported as median values and range. Categorical variables are presented as frequencies/percentages (%). Comparison between categorical variables was performed using the χ^2^ or Fisher's exact test. The Student's *t*-test was used for comparison purposes between continuous variables. Survival curves were estimated using the Kaplan-Meier method, and differences in survival were ascertained using the log-rank test.

Univariate Cox regression was performed on individual covariates to measure their marginal effect on survival. Subsequently, a multivariate Cox proportional hazards stepwise regression model was created to calculate the hazard ratios (HRs) and the corresponding 95% confidence intervals (CIs) of variables independently associated with survival. The Akaike Information Criterion (AIC) was used to select the best model for postresectional prediction of survival. The significant covariates selected by the stepwise regression were reconfirmed by a penalized Cox regression model. Model discrimination was evaluated by calculating the area under the receiver-operator-characteristic (ROC) curve (AUC) calculated for ampullary cancer overall deaths at each time point postresection. Statistical significance for all statistical calculations was defined as *P <* 0.05. Statistical analysis was performed using the R programming language (version 3.2.5, R Foundation, Vienna, Austria).

## RESULTS

### Demographic and Histopathologic Results

Overall, between January 2002 and December 2013, 111 patients underwent a pancreaticoduodenectomy for ampullary adenocarcinoma cancer. One patient was excluded from further analysis, because the death occurred in the immediate postoperative period (<30 days), leaving 110 patients as the final patient population for analysis. Patient baseline demographic and histopathological characteristics are summarized in Table 1. There were no patients lost to follow-up.

**TABLE 1:**
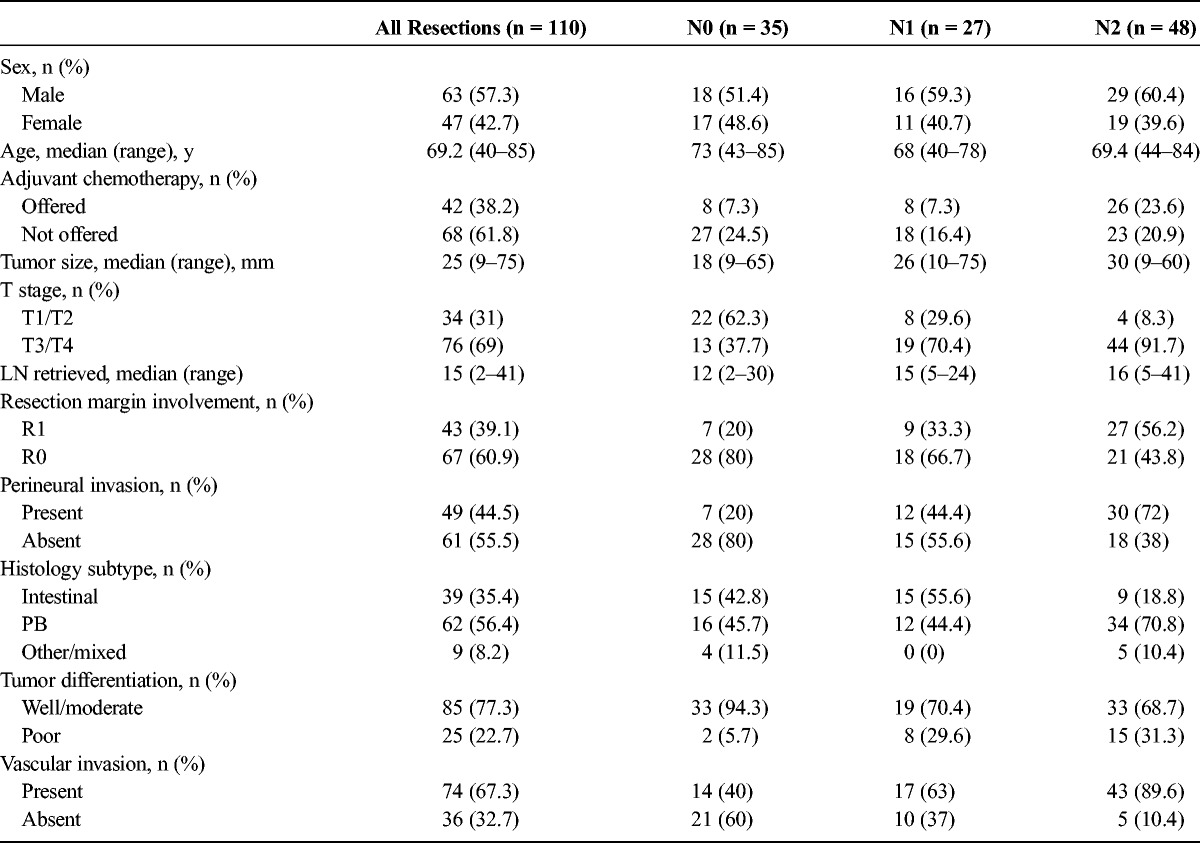
Baseline Demographic and Histologic Characteristics of the Study Population (n = 110) and the 3 LN Subgroups

The median age of the study's population was 69.2 (range, 40–85) years, and 63 (57.2%) of 110 patients were men. The median tumor diameter was 25 (range, 9–75) mm. Histological analysis revealed 34 tumors (31%) of T1 or T2 stage and 76 neoplasms (69%) of T3 or T4 stage. In 39 (35.4%) of 110 resections the tumors were of intestinal origin, in 62 cases (56.4%) were of pancreatobiliary (PB) origin, and in 9 (8.2%) were of mixed or other origin. In 85 subjects (77.3%), the tumors were well/moderately differentiated, and in 25 patients (22.7%), they were poorly differentiated. Perineural invasion was present in 49 (44.5%) of 110 resections, and evidence of vascular invasion was recorded in 74 resections (67.3%). With regards to surgical resection margin involvement, 43 (39.1%) of 110 resections were deemed as R1. In the adjuvant setting, 42 (38.2%) of 110 patients were given chemotherapy (Table 1).

### Nodal Status

The median (range) number of LNs examined per specimen was 15 (2–41). On histological analysis, a total of 75 (68.2%) of 110 specimens demonstrated evidence of LN metastases. After restaging all resections using the newly proposed nodal classification,^11^ there were 35 N0 resections (31.8%), 27 N1 resections (24.6%), and 48 N2 resections (43.6%). On univariate analysis, the proposed advanced nodal stage (N2) was significantly associated with the presence of aggressive tumor characteristics including perineural invasion (*P <* 0.001), vascular invasion (*P <* 0.001), surgical resection margin involvement (*P =* 0.02), PB subtype (*P =* 0.006), and degree of tumor differentiation (*P =* 0.01).

### LN Involvement Per Histological Subtype

**FIGURE 1:**
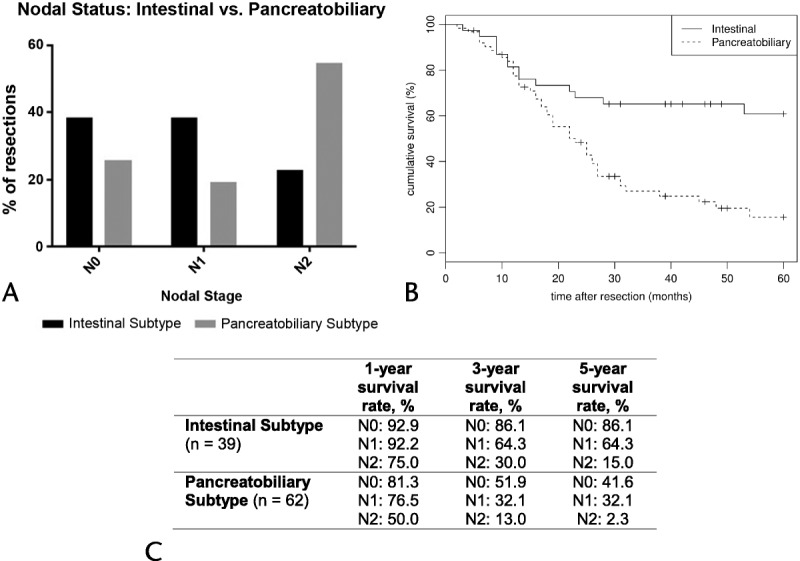
A, Nodal staging of subjects with final histology of the intestinal or PB subtype (n = 101). In the intestinal subgroup, there were 15 (38.5%) of 39 N0 resections; 15 (38.5%) of 39 N1 resections, and 9 (23%) of 39 N2 resections. The respective figures in the PB subgroup were 16 (25.8%) of 62, 12 (19.4%) of 62, and 34 (54.8%) of 62, respectively. (*P =* 0.191, 0.04, and <0.0001). B, Overall survival of patients with final histology of the intestinal and PB subtype (n = 101). The 1-, 3-, and 5-year survival rates for the intestinal subgroup were 81.5%, 62.5%, and 60.8% versus 77.4%, 27.1%, and 15.6% for the PB subgroup, respectively (log rank, *P <* 0.0001). C, The 1-, 3-, and 5-year survival rates of the 3 nodal subgroups for patients with final histology of the intestinal (n = 39) or PB subtype (n = 62).

Overall, 101 (92.7%) of 110 resected neoplasms were either of the intestinal (n = 39) or PB histological subtype (n = 62). The median number of metastatic LN was significantly lower in neoplasms of the intestinal subtype compared with lesions of the PB subtype (1.5 [0–17] and 3.0 [0–16], respectively [*P* = 0.04]). Overall, in the intestinal subgroup (n = 39), there were 15 N0 resections (38.5%), 15 N1 resections (38.5%), and 9 N2 resections (23%). The respective figures in resected specimens of the PB subtype (n = 62) were 16 N0 (25.8%), 12 N1 (19.4%), and 34 N2 (54.8%). The number of N0 resections was comparable between the 2 histological subtypes (*P =* 0.191); however, there was a significant difference in the number of N1 and N2 resections between the 2 histological subgroups (*P* values, 0.04 and <0.001, respectively; Fig. 1A).

### Postresectional Survival

**FIGURE 2:**
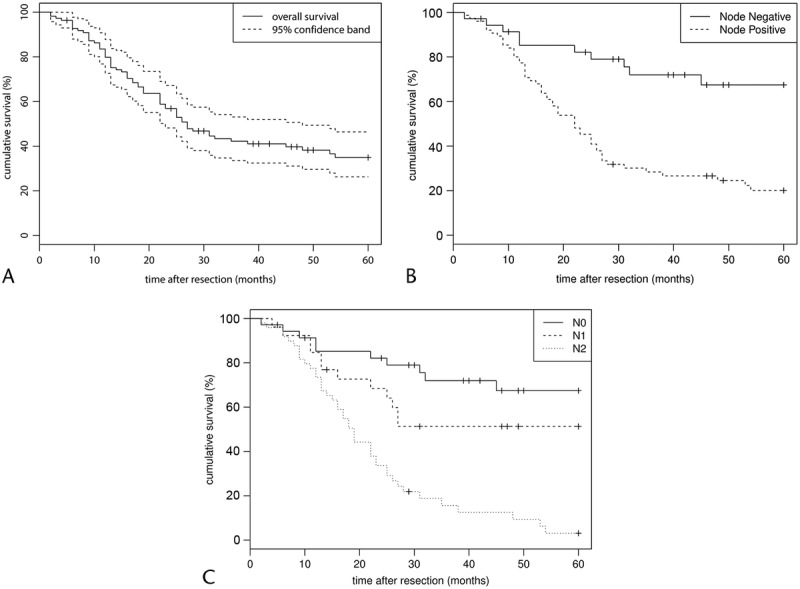
Overall postoperative survival and survival based on nodal status. A, Overall survival for the study's population (n = 110). The 1-, 3-, and 5-year survival rates were 79.8%, 42.2%, and 34.9%, respectively. B, Comparison of survival between subjects with metastatic LN versus no metastatic LN. The 1-, 3-, and 5-year survival rates for the LN metastases–positive patients were 77.0%, 27.3%, and 18.7%. The survival figures for those patients without LN metastases were 85.6%, 72.8%, and 68.5%, respectively (log rank, *P* < 0.0001). C, 1-, 3-, and 5-year survival rates of the 3 LN subgroups (N0: 85.2%, 71.9%, 67.4%; N1: 81.5%, 49.4%, 49.4%; N2: 75.0%, 19.2%, 6.4%; log rank, *P <* 0.0001).

The median duration of follow-up for the study's population (n = 110) was 37.1 (2–148) months. The overall 1-, 3-, and 5-year Kaplan-Meier estimated survival rates for the study's cohort were 79.8%, 42.2%, and 34.9%, respectively (Fig. 2A).

Overall, subjects with LN metastases had significantly worse median survival compared with subjects with no LN involvement (104 months vs 22.4 months, *P <* 0.0001). The 1-, 3-, and 5-year survival rates for patients without LN metastases were 85.6%, 72.8%, and 68.5% compared with 77.0%, 27.3%, and 18.7% in patients with LN involvement (log rank, *P <* 0.0001; Fig. 2B). After performing a subanalysis in patients with LN metastases (n = 75) as per the proposed nodal stage (N1/N2), the 1-, 3-, and 5-year survival rates were 81.5%, 49.4%, and 49.4% for N1 resections compared with 75%, 19.2%, and 6.4% for N2 resections (*P <* 0.001 [Fig. 2C]).

Additional survival analysis was performed on subjects belonging to the intestinal or PB subgroup (n = 101). The 1-, 3-, and 5-year survival rates for node-negative resections (N0) of the intestinal histological subtype had more favorable survival compared with N0 resections of the PB subtype (92.9%, 86.1%, and 86.1% vs 81.3%, 51.9%, and 41.6%, respectively; *P =* 0.032; Fig. 2D). Similarly, comparison of survival between the 2 histological subtypes for node-positive cases (N1 and N2) differed significantly in favor of the intestinal subgroup (*P =* 0.04 and 0.012, respectively; Figs. 1B, C).

### Multivariate Analysis and Prognostic Model

The following variables were entered initially in a Cox regression model: patient sex/age at time of resection, maximum tumor diameter (millimeter), tumor stage (T1/T2 or T3/T4), nodal status, histological subtype, presence/absence of vascular or perineural invasion, tumor differentiation, surgical resection margin involvement, and adjuvant chemotherapy status. The Cox proportionality assumption was validated with a χ^2^ test for Schoenfeld residuals (overall *P* value = 0.17). Preliminary analysis suggested that nodal status was a significant predictor (*P <* 0.05). To obtain a finer understanding of the dependence of hazard on nodal status, we expanded it into 3 slightly different covariates: further subclassification of nodal stage (N0/N1/N2), total number of metastatic nodes present, and ratio of metastatic to retrieved nodes (lymph node ratio [LNR]) at time of resection and included them all in the initial full model. The estimated coefficients with 95% CI in the initial Cox regression model are shown in Table 2. An AIC-guided backward stepwise model selection was subsequently performed. The selected model with the smallest AIC is also presented in Table 2. We found that the presence of vascular invasion and high LNR were significantly associated with worse survival. More precisely, the HR between patients with vascular invasion and without was 2.57 (95% CI, 1.53–4.32; *P* < 0.001). Every unit increase in LNR increases the hazard by a factor of 5.19 (95% CI, 1.20–12.5; *P* < 0.05). Because the results of stepwise regressions are known to be sensitive to the order of removal of covariates, we additionally performed an *ℓ*_1_-penalized Cox regression. The entire solution path is shown as follows, where the dashed line corresponds to the tuning parameter chosen via 10-fold cross-validation. The same 2 covariates, vascular invasion and LNR, are chosen in the cross-validated penalized Cox model, reconfirming our model selection result. A subanalysis was performed in those subjects (n = 75) with 12 or more LNs retrieved, with same 2 variables (LNR and vascular invasion) remaining as independent predictors of survival.

**TABLE 2:**
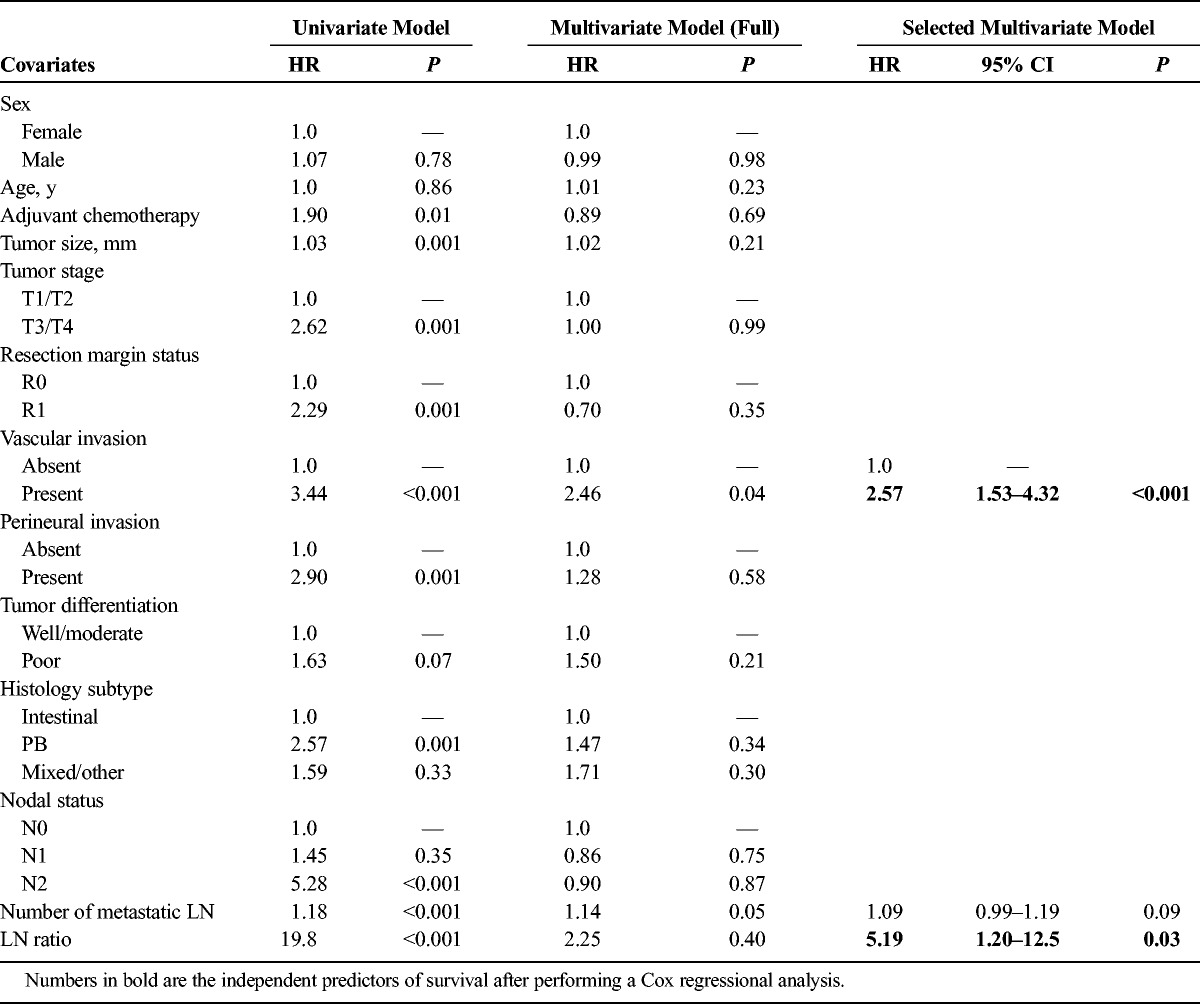
Cox Regression Analyses (Univariate/Multivariate) of Predictors of Postresectional Survival

The final multivariate Cox regression suggested the following prognosis model for prediction of postresectional survival:


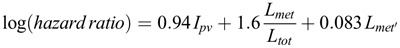


**FIGURE 3:**
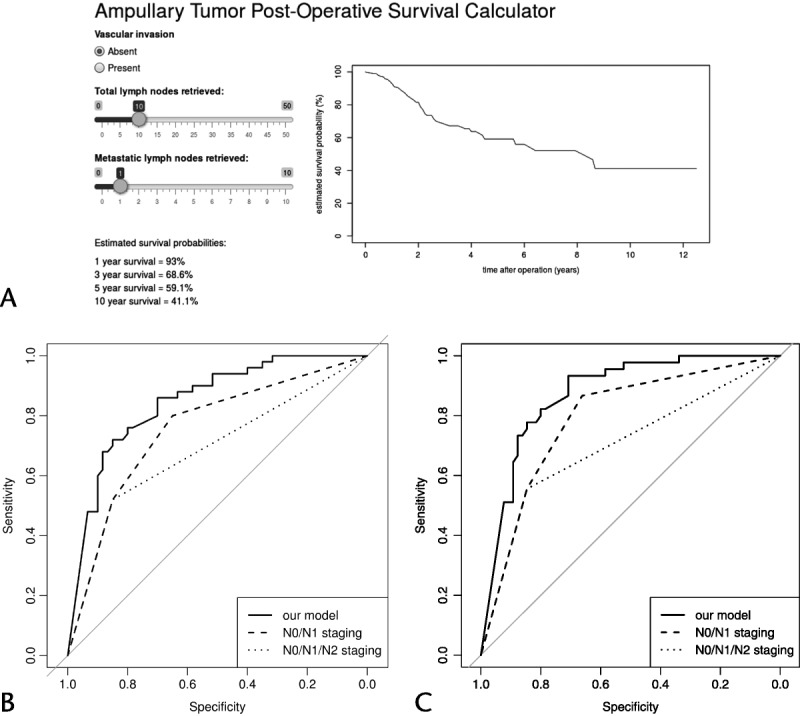
A, Snapshot of electronic survival calculator. B and C, The ROC curves for ampullary cancer overall mortality at 3 and 5 years postresection (comparison between the proposed model, the current nodal staging system [ie, NO/N1], and the recently proposed staging classification [ie, N0/N1/N2]).

Where *I*_*pv*_ = 1 if vascular invasion is present and 0 otherwise. The total number of resected and metastatic nodes is denoted by the abbreviations *L*_*met*_ and *L*_*tot*_, respectively. The previously mentioned formula has been translated to an electronic application for use by clinicians (Fig. 3A). The calculated AUCs at 3 and 5 years for our model were 0.85 and 0.87. The corresponding 3- and 5-year AUC figures for the currently used nodal staging system (ie, N0/N1) and the proposed staging system (ie, N0/N1/N2) were 0.69 and 0.70 and 0.75 and 0.79, respectively (Figs. 3B, C).

## DISCUSSION

The findings we report in this study originate from a carefully selected patient cohort, using strict histopathological criteria. Our findings demonstrate that the currently used nodal staging system (N0/N1) seems to overestimate survival in patients with 3 or more metastatic LNs. Furthermore, on the basis of the findings of a stepwise Cox regression model, using 2 independent predictors of survival (ie, LNR and vascular invasion), we have developed a pilot prognostic model. The validity of the model was strengthened by performing a subanalysis on subjects with 12 or more resected LNs. The model we propose is more sensitive than nodal status in predicting survival as our ROC curves demonstrate.

The 5-year survival rate in our study's population (34.9%) is in keeping with survival figures reported by others, adding to the validity of our patient selection criteria.^2,14,18,22^ It has been previously advocated that one of the reasons why ampullary carcinomas should be managed separately to other neoplasms arising in the close proximity of the ampulla was the more favorable survival figures reported after resection of ampullary cancer compared with other “peri-ampullary” neoplasms. Current thinking accepts that an adenoma-dysplasia-carcinoma sequence occurs on either the intestinal or biliary epithelium overlying the ampulla.^25^ In our patient population, subjects with ampullary tumors of the PB subtype behaved differently in terms of nodal disease burden and overall survival compared with resections of the intestinal subtype. Analysis of specimens of the PB subgroup revealed more advanced nodal disease compared with the intestinal subgroup as demonstrated by the nodal stage (PB: 34 N2 resections [54.8%] vs intestinal subtype: 9 N2 resections [23%], *P =* 0.002). Furthermore, the overall 5-year survival rate of 15.6% in the PB subgroup was poor and similar to postresectional survival rates for pancreatic adenocarcinoma reported elsewhere.^26^ Differences in tumor behavior, in terms of recurrence or overall survival, between the PB and intestinal subgroups have been previously reported by others.^9^ We therefore argue that histological subtype is clinically relevant to patient survival and may predict biological aggressiveness.

In the UK, the national standard on reporting nodal status in ampullary cancer as stipulated by the Royal College of Pathologists is based on the presence or absence of metastatic LN (stages N1 and N0, respectively).^24^ In 1995, Roder et al^18^ were the first to report the prognostic significance of the number of involved LN in ampullary cancer. In 2014, Kang et al^11^ proposed, in a well-defined cohort of patients, a novel substaging system for ampullary cancer based on the number of metastatic nodes. In our study population, locally advanced disease (N2 ≥3 metastatic LN) was significantly associated with adverse prognosis; however, it was not an independent predictor of survival upon multivariate analysis. Previous reports in the literature have highlighted the prognostic value of regional nodal disease either as the absolute number of metastatic nodes,^4,20^ the ratio of metastatic/retrieved nodes,^14,16^ or the anatomical LN location^12^ on survival in ampullary cancer. However, the figures reported by each study differed and in some clear description of what constitutes an ampullary neoplasm is not easily identifiable. The 2 histological factors (LNR, presence of vascular invasion) we report independently predict survival and when used in conjunction can aid clinicians in risk stratifying patients in terms of prognosis. Inadequate LN retrieval carries an increased risk of cancer understaging, and recommendations for minimum number of retrieved nodes after pancreaticoduodenectomy are in place by various professional bodies (eg, American Joint Committee in Cancer, UK Royal College of Pathologists).^24,27^ The validity of the findings we report is strengthened by the median number of LN examined per specimen (ie, 15 nodes per specimen as per the UK national standards) in our study population.

Patterns of recurrence after pancreaticoduodenectomy alone for ampullary cancer demonstrate that locoregional failure occurs in up to a third of cases.^14,27^ However, unlike the case of pancreatic cancer, the role of adjuvant chemotherapy for ampullary cancer is not clear and major work (European Study Group for Pancreatic Cancer 4 trial) is currently underway. To date, findings on survival benefit from adjuvant chemotherapy or chemoradiotherapy are not conclusive due to differing definitions of ampullary or “periampullary” tumors (study design issues/retrospective in nature/small sample size) and are therefore subjected to selection bias.^23,28–30^ Currently, patients with aggressive disease features such as metastatic LN, positive resection margins, and poorly differentiated tumors are often considered for adjuvant therapies.^23,28–30^ The prognostic model we propose, after adjusting for the chemotherapy regimes that were historically used in our cohort, is based on highly sensitive statistical analysis and seems to have higher sensitivity to the currently used nodal staging classification and is also superior to the substaging system proposed by Kang et al.^11^ Hence, we envisage that using the prognostic model, if further validated prospectively, could help clinicians to offer adjuvant therapies to those mostly in need until higher quality evidence is available.

The design of this report is retrospective; hence, bias cannot be completely eliminated from conclusion. In particular, the impact of adjuvant chemotherapy on the survival data we report should be interpreted as it is open to selection bias because the treatment was not offered as part of a standardized protocol in the 11-year study period. Nevertheless, the pilot prognostic model we propose is based on data on a carefully selected patient cohort, and although it requires validation in a larger patient population, it could aid clinicians in patient selection for adjuvant therapies in the future.
